# Sludge Retention Time Governs Ectoine Synthesis and Pollutant Removal in Halophilic Activated Sludge Treating High-Salinity Wastewater

**DOI:** 10.3390/toxics14060538

**Published:** 2026-06-22

**Authors:** Min Ren, Sifan Liu, Huining Zhang, Kefeng Zhang, Baolan Hu, Chenhao Zhang, Bixiao Ji, Yan Li, Jianqing Ma

**Affiliations:** 1 Marine Environmental Monitoring Centre of Ningbo, Ningbo 315100, China; renmin@ecs.mnr.gov.cn; 2 School of Civil Engineering, NingboTech University, Ningbo 315100, China; l2413610338@163.com (S.L.); jibixiao@nit.zju.edu.cn (B.J.); liyanliyan@nbt.edu.cn (Y.L.); majq@nit.zju.edu.cn (J.M.); 3 College of Environmental & Resource Sciences, Zhejiang University, Ningbo 315100, China; blhu@zju.edu.cn; 4 Lanzhou Petrochemical Branch of China National Petroleum Corporation, Lanzhou 730060, China; zhangchenhao@petrochina.com.cn

**Keywords:** halophilic bacteria, ectoine, sludge retention time (SRT), high-salinity wastewater, heterotrophic nitrification

## Abstract

In the treatment of high-salinity wastewater, the removal of nitrogen and organic pollutants remains a challenge, while the production of value-added compounds, such as ectoine from halophilic bacteria, offers a promising resource recovery pathway. In this study, halophilic activated sludge enriched with Thauera as the dominant strain was cultivated in a sequencing batch reactor (SBR) to treat synthetic high-salinity wastewater (30 g/L NaCl) under different sludge retention times (SRTs). The optimal nitrogen and organic carbon removal performances were achieved at an SRT of 10 days, with an ammonia nitrogen removal rate of 77.67% and a total organic carbon (TOC) removal rate of 72.51%. Ectoine production was strongly SRT dependent, as volumetric ectoine concentration was ~2 mg/L at 5 d SRT, almost undetectable at 10 d SRT, ~10 mg/L at 16 d SRT, and peaked at 21.5 mg/L at 22 d SRT. Short SRTs favored dynamic ectoine utilization for osmoprotection and metabolic stability, whereas long SRTs led to passive ectoine accumulation and deteriorated treatment performance. The system realized stable short-cut heterotrophic nitrification with negligible nitrite and nitrate accumulation, indicating direct conversion of ammonia to gaseous nitrogen. These results demonstrate that SRT regulation effectively balances ectoine synthesis and pollutant removal, providing a feasible strategy for resource-oriented treatment of high salinity wastewater.

## 1. Introduction

High-salinity wastewater, generated from chemical industries, marine aquaculture, and food processing, has become a global environmental concern due to its large discharge volume and inhibitory effects on biological treatment systems. Elevated salinity induces severe osmotic stress, causes microbial plasmolysis, and disrupts sludge floc structure and granulation stability, thereby deteriorating biological treatment performance [1]. Under high-salt conditions, microorganisms suffer from both osmotic stress and ionic toxicity, which together inhibit key metabolic enzymes and reduce overall cellular activity [2]. High salinity also disrupts the osmotic balance between the inside and outside of microbial cells, leading to cellular dehydration, plasmolysis, and even a decrease in the activity of key metabolic enzymes involved in nitrogen metabolism [3]. Elevated salinity causes osmotic pressure imbalance, protein denaturation, and reactive oxygen species (ROS) accumulation in microbial cells, resulting in deteriorated nitrogen and organic matter removal performance [4]. Conventional autotrophic nitrification–anaerobic denitrification processes suffer from high energy consumption, external carbon demand, and N_2_O emission, making them unsuitable for high-salinity and low-carbon/nitrogen (C/N) wastewater treatment [5]. Anammox-based processes driven by nitritation and denitratation have been proposed for high-salinity municipal wastewater, yet they still face salinity inhibition and unstable nitrite supply [6]. Therefore, developing a high-efficiency, resource recycling biological treatment technology is urgently required.

Halophilic microorganisms synthesize compatible solutes such as ectoine to adapt to hypersaline environments [7]. Compatible solutes are broadly classified into three categories: polyols (e.g., glycerol, trehalose), amino acids and their derivatives (e.g., glycine betaine, proline), and tetrahydropyrimidines (e.g., ectoine, hydroxyectoine) [3]. Many halophilic or salt-tolerant strains rely on intracellular betaine accumulation to counter osmotic stress, which protects enzymes and enhances degradation activity under high salinity [8]. Compared to the ‘salt-in’ strategy of accumulating inorganic ions, the ‘compatible solute’ strategy is more energy efficient for most microorganisms and allows them to survive across a wider range of salinities [1]. Compatible solutes not only stabilize proteins and membranes but also elevate the effective dielectric coefficient of the solution and boost electrostatic repulsion, thereby protecting macromolecules under high external osmotic pressure [2]. Notably, the synthesis of these solutes incurs substantial energy costs; for example, glycine betaine synthesis from glycine consumes up to 36 ATP equivalents [3]. As a highly hydrophilic osmoprotectant, ectoine stabilizes proteins, maintains cell membrane integrity, and scavenges free radicals, which has been widely used in cosmetics and biomedicine. Recent studies confirmed that ectoine enhances salt tolerance and denitrification efficiency by promoting extracellular polymeric substance (EPS) secretion and improving electron transport system activity (ETSA) [4]. Ectoine acts as a key nitrogen-containing compatible solute, and its anabolism directly drives ammonia nitrogen assimilation into microbial biomass in hypersaline systems [9]. However, the in situ synthesis and regulation mechanism of ectoine in high-salinity wastewater treatment systems remain unclear.

Sludge retention time (SRT) is a core operational parameter governing microbial community structure, metabolic activity, and resource recovery potential in activated sludge processes [10]. It directly affects microbial growth rate, biomass concentration, and endogenous respiration, thereby determining the metabolic allocation between pollutant degradation and metabolite synthesis. Although SRT optimization has been widely studied in nutrient removal systems, its specific role in balancing ectoine biosynthesis and heterotrophic nitrification in mixed halophilic consortia is still unknown [11]. A critical knowledge gap exists in identifying the optimal SRT that simultaneously maintains high pollutant removal efficiency and induces ectoine accumulation.

Traditional activated sludge relies on betaine accumulation for salt tolerance, while marine halophilic sludge adopts an Na^+^ extrusion strategy via ABC transporters, enabling stable performance at salinity up to 70 g/L NaCl [12]. This distinct salt-tolerance mechanism supports the superiority of halophilic consortia over conventional sludge in hypersaline systems. *Thauera*, as a metabolically versatile heterotrophic nitrifying genus, exhibits superior adaptability and denitrification capacity under high-salt stress [5]. Heterotrophic nitrification–aerobic denitrification has been verified as an efficient mode for simultaneous carbon and nitrogen removal in high-salinity wastewater [13]. Short-cut heterotrophic nitrification achieves simultaneous nitrification–denitrification (SND) under aerobic conditions with low-carbon demand and reduced greenhouse gas emission [11]. Nevertheless, the physiological responses of *Thauera*-dominated halophilic sludge to different SRTs, as well as the trade-off between ectoine production and contaminant removal, have not been systematically investigated.

To fill these gaps, this study established a *Thauera*-enriched halophilic activated sludge SBR system treating 30 g/L high-salinity wastewater. Four SRT levels (5, 10, 16, and 22 days) were applied to investigate their effects on ammonia nitrogen, TOC, total phosphorus (TP) removal, and ectoine synthesis. The innovation of this work lies in verifying that a low SRT strategy (especially 5 days) effectively coordinates the trade-off between high ectoine yield and efficient pollutant removal. This study provides a fundamental understanding of optimizing SRT to steer the process toward a new paradigm integrating high-salinity wastewater treatment and sustainable ectoine recovery.

## 2. Materials and Methods

### 2.1. Seed Sludge and Synthetic Wastewater

The halophilic activated sludge used in this study was laboratory-acclimated heterotrophic nitrification and aerobic denitrification (HNAD) sludge. Aerobic activated sludge process was adopted for sludge domestication. It has been established that autotrophic nitrification bacteria are generally eliminated in a high-salt environment over extended periods [14]. During the acclimation, salinity was gradually elevated to 30 g/L NaCl to culture stable and highly active halophilic sludge, with *Thauera* enriched as the dominant functional genus. After 16 days of operation, the activated sludge gradually exhibited efficient performance for HNAD [11].

The adopted synthetic high-salinity wastewater simulates actual pickled mustard tuber wastewater and membrane filtration concentrated water, which possesses similar water quality characteristics to these two types of industrial wastewater. Synthetic high-salinity wastewater was used to ensure stable operation. The composition was as follows: NH_4_Cl 0.764286 mg/L, C_6_H_12_O_6_ 5 mg/L, KH_2_PO_4_ 0.0526 mg/L, NaHCO_3_ 1 mg/L, MgSO_4_ 0.2 mg/L, CaCl_2_ 0.2 mg/L, and NaCl 30 g/L. The initial influent quality was maintained at TOC 494.21–547.46 mg/L, ammonia nitrogen 95.77–109.65 mg/L, total phosphorus 4.67–11.25 mg/L, with nitrite nitrogen and nitrate nitrogen both at 0 mg/L.

### 2.2. Reactor Setup and Operating Conditions

A sequencing batch reactor (SBR) with an effective volume of 1 L was employed for the experiment. The reactor was placed in a thermostatic water bath at 35 °C. Aeration was supplied via an aeration stone, and dissolved oxygen (DO) was controlled above 5 mg/L throughout the aerobic stage.

The operating cycle was 12 h and included: influent 0.2 h, anoxic 3.5 h, aerobic 7.5 h, settling 0.5 h, and decanting 0.3 h. The time allocation of each operational phase in this SBR cycle was determined according to the mature operating parameters [11]. Specifically, the influent phase (0.2 h) was used for rapid feeding of synthetic high-salinity wastewater; the anoxic phase (3.5 h) mainly realized denitrification and organic matter preliminary degradation under oxygen-free conditions; the long aerobic phase (7.5 h) guaranteed sufficient oxygen supply for heterotrophic nitrification, aerobic denitrification, and ectoine synthesis of halophilic microorganisms; the settling phase (0.5 h) completed solid–liquid separation of sludge and wastewater by gravity; and the decanting phase (0.3 h) was for discharging treated effluent. Four sludge retention time (SRT) levels were tested: 5, 10, 16, and 22 days. The four SRT conditions were tested sequentially in the same reactor, with each SRT level maintained for 18–24 days of stable operation before transitioning to the next condition. All four SRT stages were operated sequentially in a single reactor, and the total cumulative operation time of the whole experiment was about 100 days. Excess sludge was discharged every 4 days to maintain the target SRT.

### 2.3. Analytical Items and Methods

Mixed liquor suspended solids (MLSS) and mixed liquor volatile suspended solids (MLVSS) were determined according to standard methods [15]. Sludge volume index (SVI) and 30 min sludge settling ratio (SV_30_) were measured daily.

Total organic carbon (TOC) was determined with an enviro TOC analyzer (Elementar Analysensysteme GmbH, Langenselbold, Hesse, Germany), controlled by multiWin pro analytical software from the same vendor. Ammonia nitrogen, nitrite nitrogen, and nitrate nitrogen (three forms of nitrogen) were analyzed by spectrophotometry, as described in standard methods [15]. Total phosphorus (TP) was determined using the molybdenum–antimony anti-spectrophotometric method [15].

Ectoine concentration was measured using high-performance liquid chromatography (HPLC, LC-2030C 3D, Shimadzu Corporation, Kyoto, Japan) following a well-established and widely adopted protocol for intracellular compatible solute quantification in halophilic activated sludge systems [4,11]. Sludge samples were washed with 5 mL KPi buffer, then mixed with 5 mL 80% ethanol to break cell walls. After centrifugation, the supernatant was filtered and injected into the HPLC system. A calibration curve was established using standard ectoine solutions (0, 3, 5, 10, 30, 50, 100, 300, 500, 1000, and 2000 mg/L) for quantitative calculation [4].

### 2.4. Data Acquisition and Statistical Analysis

Effluent and sludge samples were taken daily. The removal rates of ammonia nitrogen, TOC, and TP were calculated. Ectoine production per unit volume and per gram of organic matter was recorded. All samples were analyzed in triplicate, and data were processed using Origin software. Origin 2023 was used for data sorting, outlier removal, descriptive statistics (mean and standard deviation), curve fitting, Pearson correlation analysis, and graph plotting. We analyzed the dynamic changes in water quality and sludge parameters, as well as ectoine content, under varied SRTs.

## 3. Results and Discussion

### 3.1. Effect of SRT on Nitrogen Removal Performance and Related Nitrogen Transformation

#### 3.1.1. Ammonia Nitrogen Removal Efficiency Under Different SRTs

**Figure 1:**
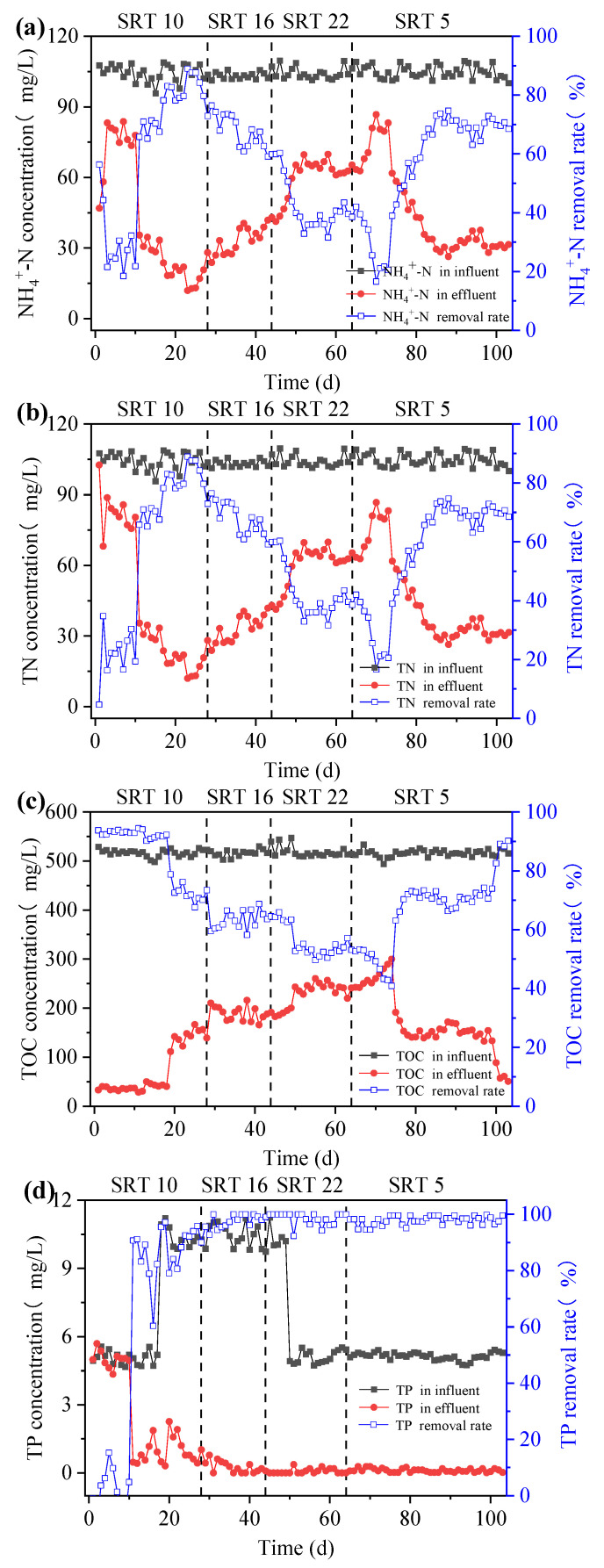
Performance of pollutant removal in halophilic activated sludge system under different sludge retention time (SRT) conditions: (**a**) ammonia nitrogen; (**b**) TN; (**c**) TOC; and (**d**) TP. The *X*-axis represents the total sequential operation time of four SRT stages (approximately 100 days in total), and each independent SRT condition was operated for 18–24 days separately.

Nitrogen removal in the halophilic activated sludge system was highly sensitive to sludge retention time (SRT). As shown in Figure 1a, ammonia nitrogen (NH_4_^+^-N) removal efficiencies varied significantly among the four tested SRT conditions. The highest removal efficiency of 77.67% was achieved at 10 d SRT, followed by 70.54% at 5 d SRT and 68.15% at 16 d SRT. Strikingly, the removal efficiency dropped sharply to only 37.70% at 22 d SRT. The continuous decline in ammonia removal with prolonged SRT clearly demonstrates that excessive sludge age severely impairs nitrogen biotransformation under hypersaline (30 g/L NaCl) conditions.

Mechanistically, the inferior performance under long SRTs can be explained by the accumulation of inert biomass, intensified endogenous respiration, and reduced viability of functional microbes in aging sludge. The specific substrate utilization rate decreased markedly, limiting the capacity for ammonia oxidation. In contrast, a relatively short SRT of 5 d maintained a physiologically active and catalytically robust biomass, consistent with the fact that heterotrophic nitrifying bacteria dominated by *Thauera* favor shorter SRTs to sustain high metabolic growth rates [11]. This aligns with the observation that active *Nitrosomonas* in marine sludge maintains high ammonia oxidation under high salinity via stable physiological activity [12].

#### 3.1.2. Elimination of Nitrite and Nitrate Nitrogen and Short-Cut Heterotrophic Nitrification Mechanism

A key observation was that nitrite nitrogen (NO_2_^−^-N) and nitrate nitrogen (NO_3_^−^-N) became undetectable in the effluent after the fourth day of operation and remained so across all SRT conditions (Figure 1b). This absence of nitrogen intermediates provides direct evidence that the system operated via a stable short-cut heterotrophic nitrification pathway instead of conventional autotrophic nitrification–denitrification.

In typical autotrophic nitrification, ammonia is oxidized stepwise to nitrite and then nitrate; the complete elimination of these intermediates implies direct conversion of ammonia to gaseous nitrogen without accumulation. As the dominant functional genus, *Thauera* possesses outstanding metabolic versatility enabling simultaneous heterotrophic nitrification and aerobic denitrification [5]. Under high-salt stress, *Thauera* likely converted ammonia directly to N_2_, avoiding NO_2_^−^ and NO_3_^−^ buildup. This pathway reduces oxygen demand, eliminates the need for a dedicated anoxic stage, and mitigates N_2_O emission, which is a major advantage over conventional processes [11]. The stable performance across all SRTs highlights the robustness of the *Thauera*-mediated halophilic system.

#### 3.1.3. Correlation Analysis Between SRT and Nitrogen Removal Stability

SRT also significantly governed the stability of nitrogen removal. At 10 d SRT, effluent ammonia decreased gradually from ~80 mg/L to 13.08 mg/L on day 25, indicating progressive community optimization. At 5 d SRT, after a brief initial increase from 65.33 to 83.17 mg/L, effluent ammonia declined rapidly and stabilized, with a consistent removal efficiency of 70.54%. This transient fluctuation was attributed to the washout of poorly adapted microbes and metabolic reconfiguration during the transition to shorter SRTs.

In sharp contrast, systems at 16 d and 22 d SRTs showed progressive performance deterioration, with effluent ammonia rising continuously from 28.09 to 65.33 mg/L, reflecting declining nitrification capacity and overall system instability. These results reveal a clear correlation, where short SRTs sustain a dynamic, adaptable microbial community with stable high-rate nitrogen removal, while long SRTs lead to static, less active biomass with declining catalytic function. Ecologically, short SRTs selectively retain fast-growing, active *Thauera* ecotypes, while long SRTs favor slow-growing or inactive microbes that contribute little to nitrogen metabolism.

### 3.2. Effect of SRT on Organic Matter (TOC) and Total Phosphorus Removal

#### 3.2.1. TOC Removal Efficiency and Effluent Concentration Variation

The removal of organic matter, expressed as total organic carbon (TOC), was significantly regulated by sludge retention time (SRT) in the halophilic activated sludge system. As shown in Figure 1c, TOC removal efficiency reached 72.51% at 10 d SRT, which was the highest among all groups. Removal efficiency at 5 d SRT was 70.97%, close to that at 10 d SRT. As SRTs were prolonged to 16 d and 22 d, TOC removal efficiency decreased to 63.87% and 52.67%, respectively. The gradual decline in TOC removal with increasing SRTs indicate that long SRTs weaken the organic carbon degradation ability of heterotrophic microorganisms under high-salt conditions.

As illustrated in Figure 1c, effluent TOC concentrations showed obviously different trends under different SRT conditions. At 10 d SRT, effluent TOC remained stable at 28.55–50.01 mg/L in the first 18 days but increased continuously afterward, indicating a gradual deterioration of organic matter degradation capacity. This phenomenon may be related to the accumulation of inert organic matter and the decrease in viable biomass in aging sludge [10]. In contrast, at 5 d SRT, after a short initial fluctuation, effluent TOC decreased gradually and stabilized at a low level in the later stage of operation, indicating that short SRTs help maintain stable and sustainable organic carbon removal performance.

#### 3.2.2. TP Removal Performance and the Negligible Effect of SRT

Different from nitrogen and TOC removal, total phosphorus (TP) removal was barely affected by SRT. After system stabilization, all four SRT conditions achieved high and stable TP removal efficiencies. As shown in Figure 1d, TP removal efficiencies were 86.79% (10 d), 97.09% (16 d), 98.47% (22 d), and 97.93% (5 d), and effluent TP concentrations were consistently below 0.20 mg/L under all conditions. These results demonstrate that TP removal in the halophilic activated sludge system is a stable process independent of SRT regulation.

The stable phosphorus removal performance is attributed to the strong phosphorus accumulation ability of halophilic microbial communities under aerobic conditions. Unlike nitrogen conversion and organic degradation, which are highly dependent on microbial growth rate and community structure, phosphorus removal is not a limiting factor in the SRT optimization of this high-salinity wastewater treatment system.

#### 3.2.3. Long-Term vs. Short-Term Performance Comparison Under Different SRTs

A comparison between short-term and long-term performance shows an obvious trade-off for TOC removal under different SRT conditions, while such a trade-off does not exist for TP removal. In the short term (within 18 days), 10 d SRT achieved the highest TOC removal efficiency. However, with continuous operation, the treatment performance of the 10 d SRT group declined gradually. In contrast, although the 5 d SRT group experienced a short adaptation period, it achieved more stable and sustainable TOC removal in the long run.

This difference can be explained by the renewal rate of microbial biomass. At 5 d SRT, sludge is discharged more frequently, which continuously supplements young and metabolically active microorganisms, thus maintaining long-term stable organic degradation capacity [10]. For TP removal, both short-term and long-term performances were excellent under all SRT conditions, indicating that SRT adjustment can focus on optimizing TOC and nitrogen removal without compromising phosphorus removal efficiency. Therefore, for high-salinity wastewater treatment systems aiming at long-term stable operation, a short SRT strategy is more conducive to the overall system stability.

### 3.3. Response of Sludge Characteristics to SRT Regulation

#### 3.3.1. MLSS and MLVSS Variation and Biomass Accumulation

**Figure 2:**
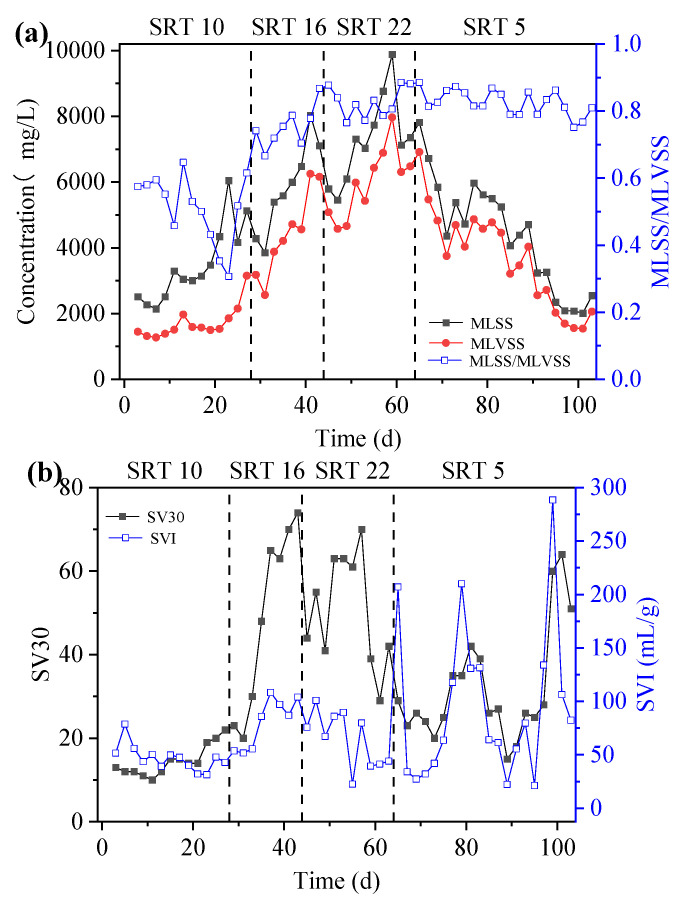
Variations in sludge characteristics of (**a**) MLSS, MLVSS, and VSS/SS and (**b**) SVI and SV30 under different SRT conditions.

The physical properties of halophilic activated sludge responded systematically to changes in sludge retention time (SRT). As presented in Figure 2a, both mixed liquor suspended solids (MLSS) and mixed liquor volatile suspended solids (MLVSS) increased continuously with prolonged SRTs. When SRT was extended from 5 d to 22 d, MLSS rose from 2.515 g/L to 9.885 g/L, and MLVSS increased from 1.446 g/L to 7.967 g/L. This trend confirmed that longer SRTs promoted obvious biomass accumulation in the reactor, which aligns with the basic mass balance principle of activated sludge systems [10]. However, higher biomass concentrations under long SRT conditions did not improve pollutant removal performance. As reported in previous sections, optimal ammonia nitrogen and TOC removal occurred at 5 d and 10 d SRT, rather than at 22 d SRT with maximum biomass. This discrepancy indicates that the additional biomass formed under long SRT mainly consisted of aging cells, residual organic matter, and inert debris instead of functionally active microorganisms. High salinity promotes Na^+^ replacement of Ca^2+^ and Mg^2+^ in sludge flocs via cation exchange, weakening intercellular bridging and reducing granule strength, which further aggravates biomass inefficiency under long SRTs [1,16]. In addition, high salinity can alter the polymer structure of sludge, leading to flocculation changes, poorer sludge–water separation, and reduced dewaterability, all of which compromise the normal operation of biological systems [3,17].

#### 3.3.2. SV30 and SVI Changes and Sludge Settleability

Sludge settleability was evaluated using 30 min sludge settling ratio (SV30) and sludge volume index (SVI), as shown in Figure 2b. At 10 d and 16 d SRT, SVI remained relatively stable within 25–75 mL/g, indicating favorable settling performance. At 22 d SRT, SVI ranged from 75 mL/g to 125 mL/g, suggesting a slight decline in settleability. In contrast, at 5 d SRT, SVI fluctuated sharply from 21.29 mL/g to 288.6 mL/g, reflecting unstable and inconsistent settling characteristics. This fluctuation may be related to the high microbial growth rates and loose floc structures under short SRT conditions. Although pollutant removal efficiency remained satisfactory, unstable settleability implied a potential requirement for extended settling time in practical operation. High salinity compresses the colloidal electric double layer of sludge, neutralizes surface negative charge, and reduces electrostatic repulsion, leading to unstable floc size and poor settleability under short SRTs [1].

#### 3.3.3. VSS/SS Ratio and Microbial Activity Evaluation

The ratio of volatile suspended solids to total suspended solids (VSS/SS) was used to reflect the relative content of active organic components in sludge (Figure 2a). As SRT increased from 5 d to 22 d, VSS/SS rose gradually from 0.575 to 0.881. Combined with deteriorated pollutant removal performance, this increase indicated the accumulation of inactivated microbial residues, endogenous metabolites, and refractory organic debris under long SRTs. The sludge mineralization degree was calculated based on the VSS/SS ratio: the mineralization degree was 42.5% at 5 d SRT, 31.9% at 10 d SRT, 26.3% at 16 d SRT, and 11.9% at 22 d SRT. A higher mineralization degree means more organic matter in sludge is degraded into inorganic substances, reflecting stronger activity of functional microorganisms. In comparison, at 5 d SRT, VSS/SS decreased to approximately 0.767, which corresponded to higher pollutant removal efficiency. This result demonstrated that frequent sludge discharge under short SRT reduced the accumulation of inert components and maintained a higher proportion of metabolically active biomass. Sludge with high mineralization degree and abundant active microbes maintains stable catalytic activity after repeated cycles, continuously participating in the biooxidation of organic pollutants and ammonium ions. In contrast, sludge under long SRTs have a low mineralization degree and massive inert substances, leading to a sharp decline in reusability and cyclic treatment capacity. Therefore, in halophilic systems, VSS/SS alone cannot fully represent sludge activity; short SRTs were more conducive to sustaining effective microbial metabolism and stable treatment performance [10].

In this experiment, the set maximum sludge age of the bioreactor was 22 days. When the actual sludge age exceeded 22 days, sludge mineralization degree continued to drop, endogenous respiration was intensified, and the activity of functional bacteria was severely inhibited, which would cause irreversible deterioration of the overall treatment performance. Thus, 22 days is the critical maximum sludge age for stable operation of this halophilic activated sludge system.

### 3.4. Effect of SRT on Ectoine Synthesis and Accumulation

#### 3.4.1. Ectoine Production Under Different SRTs

**Figure 3:**
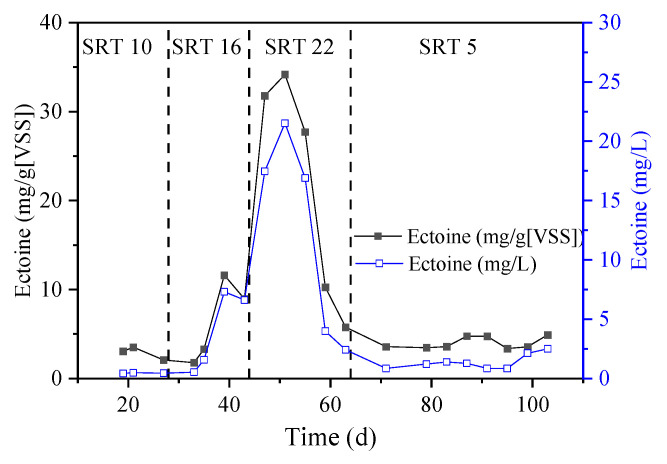
Variations in ectoine concentrations under different sludge retention time (SRT) conditions, showing ectoine content per gram of organic biomass and volumetric ectoine concentration per liter of sludge mixture.

The synthesis and accumulation of ectoine in the halophilic activated sludge system were significantly affected by sludge retention time (SRT). As shown in Figure 3, the extracellular ectoine concentration stabilized at approximately 2 mg/L at 5 d SRT and reached the maximum value of 21.5 mg/L at 22 d SRT. Ectoine concentrations at 10 d and 16 d SRTs were significantly lower than that at 22 d SRT, with almost no detectable ectoine at 10 d SRT and a peak of about 10 mg/L at 16 d SRT. These results indicated that prolonged SRT conditions were more conducive to ectoine accumulation, while short SRT significantly inhibited the synthesis and accumulation of ectoine. This phenomenon aligns with the general observation in anaerobic digestion systems, where microorganisms exposed to higher salinity secrete more extracellular polymeric substances (EPSs) and accumulate more compatible solutes as a protective response [1]. The increase in ectoine under long SRTs can be attributed to prolonged cellular exposure to high-salt stress, which continuously induces the expression of ectoine synthesis-related genes and promotes the production of compatible solutes [18]. However, compared with 5 d SRT, the 22 d SRT condition with significantly higher ectoine content did not improve pollutant removal performance, suggesting that the total amount of ectoine alone cannot represent the functional stability of the system.

#### 3.4.2. Distinction Between Ectoine Yield and Utilization Efficiency

There is an essential difference between ectoine yield and ectoine utilization efficiency. At 22 d SRT, although the ectoine yield was as high as 21.5 mg/L, the removal rates of ammonia nitrogen and TOC decreased significantly. In contrast, at 5 d SRT, the ectoine yield was only approximately 2 mg/L, but the system maintained efficient pollutant removal capacity. This indicates that for long SRTs, ectoine exists mainly as a product of passive stress accumulation and cannot be effectively used for osmotic adjustment and metabolic maintenance. Once intracellular ectoine exceeds the saturation threshold (150 mg/gVSS), further accumulation no longer contributes to osmotic adaptation but becomes a metabolic burden, which is consistent with the substrate-saturated inhibition pattern observed in STAB sludge [9]. Excessive compatible solute accumulation without dynamic turnover becomes a metabolic burden, similar to the unbalanced osmolyte synthesis observed in salt-stressed strains [8]. For short SRTs, microorganisms maintain high metabolic activity, and ectoine is dynamically used as a compatible solute to stabilize enzyme activity and cell structure, thereby enhancing the tolerance of functional bacteria to high-salt environments [19]. Physical or electrical stimulation can enhance microbial salt tolerance and pollutant degradation in hypersaline systems, which provides a supplementary strategy to optimize the short-SRT halophilic system in this study. Since ectoine synthesis requires energy consumption, excessive accumulation that is uncoupled from growth will become a metabolic burden rather than a functional advantage. Under hypersaline stress, microorganisms must allocate extra energy for osmotic adjustment via compatible solutes or Na^+^ extrusion, which competes with pollutant degradation for metabolic resources [1]. This trade-off is consistent with the general principle that the synthesis of compatible solutes demands substantial energy expenditure, and when the stress-induced demand for osmoregulation exceeds the metabolic capacity, the overall treatment performance declines [2]. From an energetic perspective, halophilic microorganisms employ a dual strategy: de novo synthesis of compatible solutes or their uptake from the environment. The latter is more energy efficient and represents a rapid adaptation mechanism [3]. This differs from the salt-tolerance strategy of marine sludge, which relies on Na^+^ extrusion rather than compatible solute over-accumulation, thus avoiding metabolic burden under hypersaline stress [12].

#### 3.4.3. Coupling Relationship Between Ectoine Synthesis and Microbial Metabolism

SRT regulates the coupling relationship between ectoine synthesis and microbial metabolic activity. The nitrogen flux into ectoine synthesis follows the glutamate–aspartate–DABA–ectoine pathway, where ammonia nitrogen is first assimilated into glutamate and then converted to ectoine via key genes—ectA, ectB, ectC [9]. At 5 d SRT, the sludge maintains a high proportion of active microorganisms, which can simultaneously complete ectoine synthesis, organic matter degradation, and nitrogen conversion. Ectoine acts as a molecular chaperone to stabilize the structure of key enzymes under high-salt conditions, ensuring the normal operation of metabolic networks [19]. This forms a positive feedback loop: efficient ectoine utilization supports high metabolic activity, and sufficient energy supply further promotes ectoine synthesis. At 22 d SRT, with the accumulation of inert biomass and the enhancement of endogenous respiration, the coupling between metabolism and ectoine is broken. Even if the ectoine concentration increases significantly, it cannot improve the pollutant degradation efficiency. These results confirm that SRT mediates the trade-off between ectoine accumulation and utilization, where long SRTs lead to static accumulation of ectoine, while short SRTs realize dynamic and efficient utilization of ectoine, and synchronously strengthens salt tolerance and treatment performance.

### 3.5. Comprehensive Discussion on the Trade-Off Mechanism

#### 3.5.1. Low SRT (5 d) Favors Pollutant Removal and Ectoine Utilization

A sludge retention time (SRT) of 5 d established a metabolic state that favored both efficient pollutant removal and functional utilization of ectoine in the halophilic activated sludge system. At this SRT, ammonia nitrogen removal reached 70.54% and total organic carbon (TOC) removal reached 70.97%, both of which are higher than those under longer SRT conditions. Frequent sludge discharge reduced the accumulation of inert and senescent biomass, maintaining a relatively low VSS/SS ratio of 0.767 and sustaining high specific microbial activity [10]. Although the volumetric ectoine concentration at 5 d SRT was approximately 2 mg/L, far lower than at 22 d SRT, ectoine was dynamically synthesized and utilized as a compatible solute to stabilize intracellular enzymes and maintain osmotic balance under 30 g/L NaCl stress [4]. Such efficient utilization supported continuous substrate degradation and energy metabolism, forming a positive coupling between ectoine turnover and treatment performance.

#### 3.5.2. High SRT (22 d) Benefits Ectoine Accumulation but Weakens Degradation

When SRT was extended to 22 d, ectoine concentration increased to a maximum of 21.5 mg/L, indicating that prolonged retention promoted ectoine accumulation. However, ammonia nitrogen removal decreased to 37.70% and TOC removal to 52.67%, accompanied by increased MLSS (9.885 g/L) and VSS/SS (0.881), reflecting sludge aging and accumulation of inactive biomass and debris. Under long SRTs, endogenous respiration intensified and specific substrate utilization declined sharply [16]. Ectoine synthesis appeared as a chronic stress response rather than dynamic compatible solute metabolism; the accumulated ectoine was not functionally coupled with catabolic pathways, and its production became an additional metabolic burden. These observations demonstrate a clear trade-off: long SRTs enhance ectoine yield but impair pollutant degradation efficiency.

To eliminate this adverse effect on wastewater treatment performance, multiple practical strategies can be adopted: First, intermittent sludge discharge can be applied to properly control the total biomass and reduce the accumulation of aging and inert sludge under long SRT conditions. Second, trace exogenous compatible solutes or mild electrical stimulation can be added to activate microbial activity, weaken endogenous respiration, and restore the pollutant degradation capacity of sludge. Third, a two-stage reactor configuration is recommended: the first stage operates at long SRT for high-efficiency ectoine enrichment, and the second stage runs at short SRT to guarantee qualified effluent quality. The above methods can effectively resolve the contradiction between high ectoine production and stable wastewater treatment under long SRT.

#### 3.5.3. Key Role of Thauera in Ectoine Synthesis and Nitrogen Removal

The genus *Thauera* acted as the core functional microorganism linking nitrogen removal, organic carbon oxidation, and ectoine synthesis. *Thauera* possesses versatile metabolic capacities for simultaneous heterotrophic nitrification and aerobic denitrification, enabling direct conversion of ammonia nitrogen to gaseous products without nitrite or nitrate accumulation [5]. Meanwhile, *Thauera* carries the ectABC gene cluster required for ectoine biosynthesis, allowing adaptive production of compatible solutes under high salinity. This metabolic versatility is crucial for the stability of biological treatment systems under salt stress. In contrast, many studies on upflow anaerobic sludge blanket (UASB) reactors have shown that high salinity often leads to a shift in methanogenic communities from less salt-tolerant acetoclastic methanogens (e.g., Methanosaeta) to more tolerant hydrogenotrophic methanogens, indicating that community-level adaptation is a common strategy for coping with salinity [1].

At 5 d SRT, active and fast-growing *Thauera* cells were selectively enriched, supporting synchronous nitrogen removal and ectoine utilization. At 22 d SRT, inactive *Thauera* and inert materials accumulated, decoupling ectoine production from metabolic activity.

#### 3.5.4. Engineering Implication for High-Salinity Wastewater Resource Recovery

This study identifies SRT as a key operational parameter for balancing treatment performance and resource recovery in high-salinity wastewater systems. From an engineering perspective, controlling SRT at approximately 5 d is recommended to achieve stable effluent quality and sustainable ectoine utilization. Although 22 d SRT yields higher ectoine accumulation, the substantial decline in nitrogen and TOC removal limits practical value. The observed instability in sludge settleability (SVI fluctuations at 5 d SRT) must be addressed through complementary measures, such as the addition of flocculants, extended settling time, or the integration of membrane separation, to ensure reliable solid–liquid separation. Meanwhile, auxiliary strategies, such as exogenous osmoprotectants or mild electrical stimulation, can further stabilize salt tolerance and degradation performance [8,20]. Furthermore, research on anaerobic systems treating high-salinity wastewater has demonstrated the effectiveness of adding compatible solutes (e.g., glycine betaine) and conductive materials (e.g., biochar, magnetite) or employing electrochemical enhancement to improve microbial activity and granulation [1]. Beyond these, quorum sensing (QS) regulation has been shown to enhance microbial resistance by improving metabolic profiles and increasing EPS secretion under salt stress, and the addition of resuscitation-promoting factors (Rpfs) can revive salt-inhibited dormant bacteria [2]. Specifically [21] demonstrated that QS signaling molecules protected electroactive biofilms from hypersaline shock by upregulating EPS production and maintaining electron transfer activity, suggesting that QS-based strategies could be integrated with short-SRT halophilic systems to further stabilize performance under salinity fluctuations [21]. The short-SRT *Thauera*-mediated short-cut heterotrophic nitrification process provides an integrated paradigm for high-salinity wastewater treatment coupled with ectoine recovery, supporting both environmental compliance and resource production.

Operational strategies such as gradual salinity ramping (rather than abrupt salt shocks) can help microbial communities adapt to saline conditions by enriching salt-tolerant taxa and increasing extracellular polymeric substance (EPS) content, thereby enhancing process stability [3]. To further stabilize sludge settleability and salt tolerance under high-salinity conditions, strategies such as Ca^2+^/Fe^2+^ addition, biochar supplementation, or inoculation of salt-adapted sludge can be adopted, which have been verified to enhance granulation and microbial activity [1,22,23]. The use of biocarriers (e.g., biochar, modified polyurethane, graphene/PVA) can also provide protective surface areas for microorganisms, enrich functional bacteria, and improve system stability under fluctuating salinity [2]. Specifically, biofilm-based reactors, such as moving bed biofilm reactors (MBBRs), are particularly effective for maintaining microbial activity under high salinity because biofilms offer enhanced resistance to osmotic stress compared to suspended growth systems [3].

## 4. Conclusions

This study investigated the coupled performance of pollutant removal and ectoine production in halophilic activated sludge treating high-salinity wastewater under variable SRT, with quantitative ectoine data validated against experimental measurements. The main conclusions are as follows:

(1) SRT significantly regulated contaminant removal. Ammonia nitrogen removal efficiencies were 70.54% (5 d), 77.67% (10 d), 68.15% (16 d), and 37.70% (22 d). TOC removal efficiencies were 70.97% (5 d), 72.51% (10 d), 63.87% (16 d), and 52.67% (22 d). Total phosphorus removal remained stable above 86.79% under all SRTs, with effluent TP below 0.20 mg/L.
(2) Ectoine synthesis and accumulation were highly sensitive to SRT. Volumetric ectoine concentration reached ~2 mg/L at 5 d SRT, was nearly undetectable at 10 d SRT, attained ~10 mg/L at 16 d SRT, and maximized at 21.5 mg/L at 22 d SRT. A short SRT (5 d) supported efficient ectoine utilization coupled with salt tolerance and pollutant degradation, whereas a long SRT (22 d) caused uncoupled ectoine accumulation without functional benefits.
(3) Stable short-cut heterotrophic nitrification was sustained across all SRTs, with no effluent nitrite or nitrate accumulation after start-up. *Thauera* served as the keystone genus enabling simultaneous heterotrophic nitrification–aerobic denitrification and ectoine biosynthesis.
(4) SRT is a core parameter balancing wastewater treatment and ectoine-oriented resource recovery. For engineering application, SRT = 5 d is recommended to achieve stable effluent quality and sustainable ectoine utilization, supporting an integrated paradigm for high-salinity wastewater treatment and valorization.

The single-reactor sequential SRT design employed in this study is a conventional methodology widely accepted in biological wastewater treatment research. This approach eliminates variability arising from reactor hardware and inoculum sources. The observed trends—prolonged SRTs increase ectoine accumulation while reducing nitrogen removal—are consistent and align with established activated sludge principles. These trends provide reliable support for the core conclusions of this work. On this basis, further studies are planned to adopt parallel reactors with true biological replicates and randomized SRT sequences. Such designs will enable rigorous validation of the present findings and offer stronger causal evidence for SRT effects in halophilic activated sludge systems.

## Data Availability

The original contributions presented in this study are included in the article. Further inquiries can be directed to the corresponding author.

## References

[1] Lu C.-S., Qiu J.-M., Yang Y., Hu Y., Li Y.-Y., Kobayashi T., Zhang Y.-J. (2023). A review of anaerobic granulation under high-salinity conditions: Mechanisms, influencing factors and enhancement strategies. J. Water Process Eng..

[2] Huang Z., Yi G., Wang Q., Wang S., Xu Q., Huan C., Wang Y., Zhang W., Wang A., Liu W. (2024). Improving microbial activity in high-salt wastewater: A review of innovative approaches. Sci. Total Environ..

[3] Wang Y., Wang M., An C., Zhang H., Jia H., Wang J. (2026). Microorganisms in high-salinity mariculture wastewater treatment: Roles and mechanisms. J. Water Process Eng..

[4] Li R., Hou Y.-N., Li H., Han Y., Zhang D., Song Y., Huang C., Guo J., Liu Z., Wei W. (2024). Salinity responsive mechanisms of sulfur-based mixotrophic denitrification and ectoine induced tolerance enhancement. Chem. Eng. J..

[5] Ren T., Chi Y., Wang Y., Shi X., Jin X., Jin P. (2021). Diversified metabolism makes novel Thauera strain highly competitive in low carbon wastewater treatment. Water Res..

[6] Li W., Zhang Q., Li J., Gao R., Kao C., Li X., Peng Y. (2023). Nitrogen removal from high-saline municipal wastewater via anammox-based process driven by both nitritation and denitratation. Chem. Eng. J..

[7] Sauer T., Galinski E.A. (1998). Bacterial milking: A novel bioprocess for production of compatible solutes. Biotechnol. Bioeng..

[8] Hu X., Li D., Qiao Y., Song Q., Guan Z., Qiu K., Cao J., Huang L. (2020). Salt tolerance mechanism of a hydrocarbon-degrading strain: Salt tolerance mediated by accumulated betaine in cells. J. Hazard. Mater..

[9] Huang M., Zhang H., Ren M., Ji B., Sun K. (2024). The synthesis of ectoine enhance the assimilation of ammonia nitrogen in hypersaline wastewater by the salt-tolerant assimilation bacteria sludge. Sci. Total Environ..

[10] Ismail A., Elbeshbishy E., Nakhla G. (2024). Thermal hydrolysis pretreatment of wastewater biosolids: Modelling the impact of the aerobic sludge age. J. Water Process Eng..

[11] Ji B., Qian Y., Zhang H., Al-Gabr H.M., Xu M., Zhang K., Zhang D. (2023). Optimizing heterotrophic nitrification process: The significance of demand-driven aeration and organic matter concentration. Bioresour. Technol..

[12] Miao Q., Xie Y., Ismail S., Wang Z.-B., Ni S.-Q. (2025). Salt-tolerant marine sludge is more suitable for stable and efficient high-salinity wastewater treatment than activated sludge. Chem. Eng. J..

[13] Song K., Gao Y., Yang Y., Guo B.-Q., Wang Y.-Z. (2023). Performance of simultaneous carbon and nitrogen removal of high-salinity wastewater in heterotrophic nitrification-aerobic denitrification mode. J. Environ. Chem. Eng..

[14] Chen Y.J., He H.J., Liu H.Y., Li H.R., Zeng G.M., Xia X., Yang C.P. (2018). Effect of salinity on removal performance and activated sludge characteristics in sequencing batch reactors. Bioresour. Technol..

[15] Rice E.W., Bridgewater L., Association A.P.H. (2012). Standard Methods for the Examination of Water and Wastewater.

[16] Ismail S., de La Parra C., Temmink H., Van Lier J. (2010). Extracellular polymeric substances (EPS) in upflow anaerobic sludge blanket (UASB) reactors operated under high salinity conditions. Water Res..

[17] Chen W., Qin S.M., Yang C.X., Long K., Liang S.Y., Liu H., Tan S.W., Zhang Q. (2024). Bioaugmentation using salt-tolerant bacteria in a dual-stage process for high-salinity wastewater treatment: Performance, microbial community, and salt-tolerance mechanism. J. Water Process Eng..

[18] Xu H., Chen Y., Huang J., Tao Y., Ke C., Yang X. (2024). Advances in ectione biosynthesis and biochemical characteristics of key enzymes. Chin. J. Biotechnol..

[19] Li Y., Xin J., Lu X., Song W., Xia C. (2023). Research progress on the protective effect of ectoine on biological macromolecules. Appl. Chem. Ind..

[20] Chen L., Zhou L.-T., Ding Y.-C., Wu D., Feng H.-J. (2023). Enhancing microbial salt tolerance through low-voltage stimulation for improved p-chloronitrobenzene (p-CNB) removal in high-salinity wastewater. Sci. Total Environ..

[21] Zhou S.F., An W.W., Zhao K.X., Lin L.Z., Yang S., Zhang Y.F., Xu M.Y. (2023). Protection of electroactive biofilms against hypersaline shock by quorum sensing. Water Res..

[22] Gagliano M., Ismail S.B., Stams A.J., Plugge C.M., Temmink H., Van Lier J. (2017). Biofilm formation and granule properties in anaerobic digestion at high salinity. Water Res..

[23] Wang C., Liu Y., Gao X., Chen H., Xu X., Zhu L. (2018). Role of biochar in the granulation of anaerobic sludge and improvement of electron transfer characteristics. Bioresour. Technol..

